# Verbal and visual-spatial working memory and mathematical ability in different domains throughout primary school

**DOI:** 10.3758/s13421-014-0480-4

**Published:** 2014-11-07

**Authors:** Eva Van de Weijer-Bergsma, Evelyn H. Kroesbergen, Johannes E. H. Van Luit

**Affiliations:** Faculty of Social and Behavioral Sciences, Department of Pedagogical and Educational Sciences, Utrecht University, P.O. Box 80140, 3508 TC Utrecht, The Netherlands

**Keywords:** Visual spatial, Verbal, Working memory, Math ability, Children

## Abstract

The relative importance of visual-spatial and verbal working memory for mathematics performance and learning seems to vary with age, the novelty of the material, and the specific math domain that is investigated. In this study, the relations between verbal and visual-spatial working memory and performance in four math domains (i.e., addition, subtraction, multiplication, and division) at different ages during primary school are investigated. Children (*N* = 4337) from grades 2 through 6 participated. Visual-spatial and verbal working memory were assessed using online computerized tasks. Math performance was assessed at the start, middle, and end of the school year using a speeded arithmetic test. Multilevel Multigroup Latent Growth Modeling was used to model individual differences in level and growth in math performance, and examine the predictive value of working memory per grade, while controlling for effects of classroom membership. The results showed that as grade level progressed, the predictive value of visual-spatial working memory for individual differences in level of mathematics performance waned, while the predictive value of verbal working memory increased. Working memory did not predict individual differences between children in their rate of performance growth throughout the school year. These findings are discussed in relation to three, not mutually exclusive, explanations for such age-related findings.

To solve a math problem, such as 7 × 12, a child needs to hold the relevant information in mind and manipulate this information. For example, this problem may be solved by the strategy to split this problem into subproblems (e.g., 7 × 10, 7 × 2 is 70 + 14), requiring the child to keep the answer to one of the subproblems in mind, while solving the second one, and adding the outcomes to produce the answer to the original problem. In addition or subtraction calculations with multiple digits, such as 27 + 59 or 47 −19, carrying or borrowing of a digit from one column to another also requires a child to keep track of the manipulations and intermediate solutions. Indeed, there is ample of evidence that children with a higher working memory capacity have an advantage in mathematics (Friso-van den Bos, Van der Ven, Kroesbergen, & Van Luit, 2013; Raghubar, Barnes, & Hecht, 2010).

The most widely used model of working memory (WM) includes several components: the central executive, phonological loop, visual-spatial sketchpad, and episodic buffer (Baddeley, 1986, 2000). The central executive is a domain-general, attentional control system involved in several processes such as the selection and execution of strategies, retrieval of information from long term memory, monitoring of input, simultaneously storing and processing of information, and the coordination of the other components of the WM system. The visual-spatial sketchpad involves temporary storage and rehearsal of visual and spatial information, while the phonological loop involves storage and rehearsal of phonological and auditory information. The episodic buffer – a temporary storage system that is responsible for the integration of information from a variety of sources – is the third slave system (Baddeley, 2000). Functioning of the two domain-specific slave systems is usually measured using simple span tasks, in which increasingly longer strings of information are immediately recalled without further processing. Functioning of the central executive is traditionally measured with complex span tasks, in which storage as well as processing or manipulation of information is required (Kail & Hall, 2001). In other words, working memory can be distinguished from short-term memory, which only involves the temporary storage of information by the slave systems, whereas working memory involves storage as well as processing of information. Although the central executive is a domain-general component, the tasks used to measure its functioning also tap into one (or both) of the domain-specific slave storage systems. The multicomponent nature of this model allows researchers to examine whether the use of different subcomponents in mathematics vary as a function of the type of math test, age, individual differences in ability level, and the type of strategy used (Raghubar et al., 2010).

The central executive, as well as the visual-spatial sketchpad and the phonological loop have been shown to be associated with mathematics performance and learning in children (Alloway & Alloway, 2010; Bull, Espy, & Wiebe, 2008; De Smedt et al., 2009; Friso-van den Bos et al., 2013; Geary, 2011; Geary, Hoard, Byrd-Craven, & Catherine DeSoto, 2004; Holmes & Adams, 2006; Imbo & Vandierendonck, 2007; Meyer, Salimpoor, Wu, Geary, & Menon, 2010; Raghubar et al., 2010; Swanson & Beebe-Frankenberger, 2004; Swanson, 2006; Toll, Van der Ven, Kroesbergen, & Van Luit, 2011; Van der Ven, Van der Maas, Straatemeier, & Jansen, 2013). However, the strength of the relationship between different working memory modalities and mathematics performance is found to vary as a result of the type of mathematics tests used, and the strategies and mental models these tests elicit. In their recent meta-analysis, Friso-van den Bos and colleagues (2013) found the majority of working memory components to be more strongly associated with general mathematics tests, such as a national curriculum test and composite measures, than with purely arithmetical measures. General mathematics tests often include a broad variety of problem types, requiring children to switch between operations, strategies, and mental models. Nevertheless, even solving basic arithmetic problems may elicit both visual-spatial and verbal representations and strategies (Imbo & LeFevre, 2010; Logie, Gilhooly, & Wynn, 1994). So, since solving mathematical problems may elicit visual-spatial as well as verbal representations and strategies, both visual-spatial and verbal working memory components are likely to be involved in learning mathematics.

Several studies indicate that the relationship between working memory and mathematics changes with age (Andersson & Lyxell, 2007; De Smedt et al., 2009; Henry & MacLean, 2003; Holmes & Adams, 2006; Kyttälä, Aunio, & Hautamäki, 2010; McKenzie, Bull, & Gray, 2003; Rasmussen & Bisanz, 2005; Van der Ven et al., 2013). The results from studies during preschool, primary school, and adolescence suggest that younger children rely more on visual-spatial working memory when learning and applying new mathematical skills, whereas older children rely more on verbal working memory after skills have been learned.

Van der Ven et al. (2013) introduced three, not mutually exclusive, explanations for the decrease in the relationship between visual-spatial working memory and math performance as children grow older. First, according to the *developmental* explanation, younger children rely more on visual-spatial representations (e.g., number lines) and use more visual-spatial strategies (e.g., finger counting) (De Smedt et al., 2009; Geary et al., 2004). As children grow older, however, and associations between math problems and their answers become verbally memorized (partly due to rote-learning in school), they rely on more verbal strategies and representations (De Smedt et al., 2009; Holmes & Adams, 2006). Second, the *novelty* explanation supposes the shift from visual-spatial to verbal strategies is caused by the novelty of the material. This explanation assumes that children of any age, as well as adults, may rely on visual-spatial working memory when presented with novel and challenging math problems (Tronsky, 2005). On the other hand, research also indicates that working memory involvement diminishes when associations between math problems and their answers are automatized and direct retrieval is used (Ackerman, 1988; Ackerman & Gianciolo, 2000; Geary et al., 2004; Imbo & Vandierendonck, 2007). Third, the *[math] domain specificity* explanation predicts the relation between math and visual-spatial working memory differs between math domains. According to Van der Ven and colleagues (2013), addition and subtraction may be performed by manipulation or visualization of the manipulation, while multiplication and division problems are more often solved by retrieving verbally memorized facts. Van der Ven et al. (2013) investigated these three explanations in a large sample of primary school children using an adaptive computerized test to assess visual-spatial working memory and arithmetic abilities. Although their results show a significant relationship between visual-spatial working memory and all four different math domains, the strength of the relationship differed with age and across math domains. Addition and subtraction showed the strongest relationship with visual-spatial working memory, while the relationship with multiplication and division was smaller, supporting the math domain specificity explanation. In addition and subtraction, but not in multiplication and division, the relationship with visual-spatial working memory tended to decrease with age, supporting the developmental explanation in the first two domains. The finding that the relationship between visual-spatial working memory and the different domains shows a peak at the grades in which the domain is introduced as part of the school math curriculum also provides some evidence for the novelty explanation. Although these results support the notion that strategies in addition and subtraction are later replaced by verbal strategies and retrieval, Van der Ven et al. (2013) emphasize that mere rote learning could not explain their findings. As a result of their use of an adaptive testing procedure, children were never presented with problems they would have memorized, because these problems would be too easy. In multiplication, verbal and retrieval strategies may already have been more important from the beginning, since many schools apply verbal rote memorization to learn multiplication facts. However, since Van der Ven et al. (2013) did not include a measure of verbal working memory, it remains uncertain how the relationship between verbal working memory and the different math domains changes with age. To the best of our knowledge, it has also not been examined how visual-spatial and verbal working memory may be differentially related to growth in math performance during the school year(s). Knowledge on this topic may be important for the development of mathematics curriculum and instruction, for example by modifying instructions and activities to reduce working memory load or by helping children develop different strategies based on their developmental stage or individual needs.

## Goals of the present study

To summarize, both visual-spatial and verbal working memory seem to be involved in math abilities. However, the relative contribution of visual-spatial and verbal working memory to the development of math abilities seems to change with age. Previous studies have not yet examined this in a systematic way, including relatively small samples and a limited age range (Andersson & Lyxell, 2007; De Smedt et al., 2009; Henry & MacLean, 2003; Holmes & Adams, 2006; Kyttälä et al., 2010; Meyer et al., 2010; Rasmussen & Bisanz, 2005), or lacking a measure of verbal working memory (Van der Ven et al., 2013). To investigate how these relationships change as children go through primary school, we need to incorporate both visual-spatial and verbal working memory tasks and examine large samples from different grades.

Our aim is to provide a systematic examination of developmental changes in the strength of the relationship between visual-spatial and verbal working memory on the one hand and children’s math ability in the four basic mathematical domains on the other hand (i.e., addition, subtraction, multiplication and division). We examine the relationship using a repeated-measures design with three measurements of domain-specific math abilities during the school year, in grades 2 through 6. Based on the literature, we have several expectations. First, we expect visual-spatial and verbal working memory to be related to performance in all four domains of mathematics (Friso-van den Bos et al., 2013). Second, we expect visual-spatial working memory to be more strongly related to initial performance at the start of the school year, and verbal working memory more strongly related to developmental rate of change during the school year (Geary et al., 2004; Imbo & Vandierendonck, 2007). Third, the relationship between visual-spatial working memory and math performance is expected to be strongest for addition and subtraction compared to multiplication and division, while the reverse is expected for verbal working memory (Van der Ven et al., 2013). Finally, we expect the strength of the relationship between visual-spatial working memory and math performance to decrease in higher grades and the strength of the relationship between verbal working memory and math performance to increase in higher grades (e.g., Friso-van den Bos et al., 2013; Raghubar et al., 2010; Van der Ven et al., 2013).

## Method

### Participants

Data used in this study were collected as part of a large-scale intervention study on the effects of teacher training in differentiated math education on student math performance. A total of 4,337 children (grades 2 through 6) from 32 elementary schools in The Netherlands participated. Children came from 185 classes, of which 47 classes were multigrade classes consisting of children from two (e.g., grade 3 and 4) or sometimes three grades. Parents received written information about the study and we used a passive informed consent procedure. Parents informed the teacher of their child or a designated contact person at their school when they did not want their child to participate. The study was approved by the ethics committee of the Faculty of Social and Behavioral Science, Utrecht University.

### Materials

#### Working memory

Two online computerized working memory tasks suitable for self-reliant administration in the classroom were administered, the Lion game and the Monkey game. The Lion game is a visual-spatial complex span task, in which children have to search for colored lions (Van de Weijer-Bergsma, Kroesbergen, Prast, & Van Luit, 2014). Children are presented with a 4 × 4 matrix containing 16 bushes. In each trial, eight lions of different colors (red, blue, green, yellow, purple) are consecutively presented at different locations in the matrix for 2000 ms. Children have to remember the last location where a lion of a certain color (e.g., red) has appeared and use the mouse button to click on that location after the sequence has ended. The task consists of five levels each of four items, in which working memory load is manipulated by the number of colors – and hence, the number of locations – children have to remember and update. No cut-off rules were applied; all children finished all 20 items. We scored the proportion of items recalled in the correct location. The Lion game has excellent internal consistency (Cronbach’s α between .86 and .90), satisfactory test-retest reliability (*ρ* = .71), and good concurrent and predictive validity (Van de Weijer-Bergsma et al., 2014).

The Monkey game is a verbal span-backwards task, in which children have to remember and recall different words backward. Children hear spoken words (i.e., moon, fish, rose, eye, house, ice, fire, cat, coat). In Dutch, these words (i.e, maan, vis, roos, oog, huis, ijs, vuur, poes, jas) are some of the words first learned in reading by children in the first grade. Children have to remember the words and recall them backwards, by clicking on the written words presented visually in a 3 × 3 matrix. The task consists of five levels each of four items, in which working memory load is manipulated by the number of words children have to remember and recall backward, ranging from two words in level 1 to six words in level 5. No cut-off rules were applied; all children finished all 20 items. We scored the proportion of items recalled in the correct order. The Monkey game has excellent internal consistency (Cronbach’s α between .78 and .89) and shows good concurrent and predictive validity (Van de Weijer-Bergsma, Kroesbergen, & Van Luit, 2014). Several studies have shown that backward span tasks require executive processing in children, and can therefore be considered a measure of working memory during childhood (Alloway, Gathercole, & Pickering, 2006; Gathercole, Brown, & Pickering, 2003).

#### Math fluency

The Arithmetic Tempo Test (ATT; De Vos, 1992) is a standardized paper-and-pencil test frequently used in Dutch and Flemish education to measure math fluency. Its psychometric value has been established in a sample of 10,059 Flemish children (Ghesquière & Ruijssenaars, 1994). Five sets of 40 formal math problems are presented, respectively in the domains of addition (+), subtraction (−), multiplication (×), division (÷), and a mixture of the four domains (+, −, ×, ÷). In each set, children have to solve as many problems as possible within 1 minute. All problems consist of two-operand equations with an outcome smaller than 100 and both operands ranging between 0 and 90. The total number of problems answered correctly for each domain was used as a domain score. In our study, test-retest reliability for the different domains ranged from *ρ =* .84 to .87 (one-sided *p* < .001) after 4 months, and from *ρ =* .82 to .86 (one-sided *p* < .001) after 8 months. Additionally, the total number of solved problems was strongly associated with performance on a national mathematics test consisting of primarily context problems (*ρ* = .74, one-sided *p* < .001). A principal component analyses showed that, in each grade and at each measurement, performance of the four domains loaded onto a single factor. The variance explained by this factor, which we call ‘math fluency,’ varied between 59.5 % (in grade 2, where only addition and subtraction were administered) and 79.8 % (in grade 6, where all four domains were administered).

### Procedure

Measurements took place on three occasions during the school year of 2012–2013: in September–October 2012 (T1), in January–February 2013 (T2), and in May–June 2013 (T3). At T1, visual-spatial WM was assessed using the Lion game in grades 2 through 6. Teachers received an e-mail containing login information for their class of children and were asked to let all students finish the task within a period of three weeks. At T2, we assessed verbal WM using the Monkey game in grades 2 through 6. The ATT was administered by the teacher at T1, T2, and T3 in grades 2 through 6. Children in grade 2 finished only the first two ATT columns since multiplication is introduced only later during the school year of grade 2 and division is introduced in grade 3. Children from grades 3 through 6 finished all five ATT columns.

#### Missing values

Because of the large scale of the study, no information about missing data was collected. For the ATT, 53 children (13, 17, 10, 6, and 7 children from grades 2, 3, 4, 5, and 6, respectively) had missing data on all three measurements. Most of these children changed schools during the study. We used ATT data for the remaining 4,285 children in the analyses (see Table 1 for sample characteristics), with 74 % of children providing data on all three measurements, 22 % of children providing data for two measurements, and 4 % of children providing data for only one measurement. Data in most cases were missing due to absence from school (e.g., in case of sickness) at the time of assessment. In three cases, children failed to make one or two of the ATT columns for unknown reasons. Of the 4,285 children, a total number of 3,830 children (89 %) provided data on visual-spatial working memory, while 3,499 children (82 %) provided data on verbal working memory, of which 3,234 children (75 %) provided both.

**Table 1:** Sample characteristics

	*n*	% of boys
Grade 2	835	54.1
Grade 3	836	51.1
Grade 4	853	48.9
Grade 5	848	49.9
Grade 6	913	49.7
Total	4,285	50.7

#### Data-analysis

In step 1, we fitted four overall (i.e., for the whole sample) Univariate Latent Growth Curve Models to investigate the level and growth rate of math fluency in each domain (i.e., addition, subtraction, multiplication, and division) as well as the association between level and growth rate. The Mplus statistical package (Version 7; Muthén & Muthén, 1998-2010) was used. A full estimation maximum likelihood (MLR) method was applied, since it is robust to non-normality and can handle missing data. Usually in linear growth models, regressions weights for T1, T2, and T3 are fixed at 0, 1, and 2, and as a result, the intercept is mainly estimated based on T1. However, since one of our predictors (i.e., verbal working memory at T2) cannot be used to predict past performance at T1, we used a centered growth model. In each model, measurement at T2 was chosen as time point zero, and regression weights for the slope were fixed at −1 for the measurement at T1 and 1 for the measurement at T2. As a result, level of performance is mainly estimated based on T2 performance, but also influenced by T1 and T3 performance. Although no attempt was made to explain variance at the classroom level, in all models the standard errors were corrected for the nested structure using an automatic multilevel modeling setup (Stapleton, 2006). Applying the Mplus statement “type is complex” ensures that part of the model-variance is attributed to between-class variance (i.e., variance in achievement outcome existing between classrooms) rather than only to within-classroom variance. We evaluated model fit with the comparative fit index (CFI), Tucker-Lewis index (TLI), and root mean square error of approximation (RMSEA). CFI and TLI are good if > .95 and acceptable if > .90. RMSEA is good if < 0.05 and acceptable if ≤ 0.80 (Browne & Cudeck, 1993). Because of the large sample size however, we expect the χ^2^ tests to be significant.

In step 2, after determining which type of overall growth model fitted the data best, we performed Multigroup Latent Growth Curve analyses on the univariate models with a within-level grouping command to estimate parameters (intercept and slope) for each grade separately (i.e., grades 2, 3, 4, 5, and 6 for addition and subtraction, grades 3, 4, 5, and 6 for multiplication and division). We used a within-level grouping command, since some classes are multigrade classes (Muthen & Asparouhov, 2011). In these multigroup analyses, we allowed intercept and slope means and variances to be freely estimated for each grade. We used Wald χ^2^ tests to test for differences in parameter values between the two highest and lowest grades (i.e., grade 2 and 6 or grade 3 and 6) only, to limit the number of comparisons.

In step 3, we extended the four overall models from step 1 with visual-spatial and verbal working memory as predictors to test whether individual differences in the level of performance (intercept) and the rate of growth (slope) in math fluency were predicted by working memory, and whether these models provided a good fit. Also, visual-spatial and verbal working memory were allowed to co-vary.

In step 4, a within-level grouping command (i.e., grade) was added again to examine age-related differences in the predictive value of visual-spatial and verbal working memory. In this step of the analyses, working memory was only regressed on intercepts and slopes in those grades with significant variation in step 2. Steiger’s Z (*Z*_*H*_) was used to test if differences within grades in dependent standardized estimates of visual-spatial versus verbal working memory are statistically significant (Steiger, 1980; Hoerger, 2013), taking into account the covariance between the two working memory tasks. To test whether differences in independent standardized estimates and covariances between the lowest and highest grades are statistically significant, we used the Fisher *r*-to-*z* transformation (Lowry, 2013; Steiger, 1980).

When multiple comparisons are made (in step 2 and 4), Holm’s correction was applied to ensure that the chance for a Type I error did not exceed the .05 level. In Holm’s procedure, first the *p*-values of the relevant test outcomes are ranked from the smallest to the largest. The smallest outcome *p*-value needs to be smaller or equal to α/k (where α = .05 and k is the number of tests). The second smallest *p*-value is compared to α/(k-1). This sequence is followed until a corrected *p*-value becomes larger than .05. For example, when three comparisons are made, testing at the .05 level, in order to be able to speak of a significant difference, the smallest initial *p*-value needs to be ≤ .017, while the second smallest *p*-level needs to be ≤ .025, and the final *p*-value needs to be ≤ .05 (Holm, 1979).

## Results

Descriptive results for visual-spatial and verbal working memory and math fluency in the four domains are presented in Table 2. No univariate or multivariate outliers were identified using Z-scores and mahalanobis distances, respectively. Normality of distributions of the variables was examined by calculating the standardized skewness and kurtosis index (statistic divided by standard error). Skewness and kurtosis indices were found to be higher than 3 for the Lion game (−17.8 and 4.5, respectively) and the Monkey game (−23.9 and 4.0, respectively) scores, indicating that the distributions differed significantly from normal. Skewness and kurtosis indices for the ATT domain scores were also sometimes found to be higher than 3 (values ranging from 0.1 to 7.3). Non-normality was taken into account in all statistical analyses.

**Table 2:** Descriptives for the domains of the Arithmetic Tempo Test and Working Memory tasks for grades 2 through 6

		Grade 2			Grade 3			Grade 4			Grade 5			Grade 6		
		*n*	mean	SD	*n*	mean	SD	*n*	mean	SD	n	mean	SD	n	mean	SD
Addition	T1	777	14.87	4.45	789	18.64	5.15	769	21.70	4.94	793	25.07	4.61	836	27.67	4.75
	T2	748	16.46	4.78	769	19.79	4.89	823	23.69	4.83	824	26.55	4.97	847	28.86	4.64
	T3	704	17.33	5.09	686	21.07	5.33	719	24.60	4.88	709	27.23	4.73	764	29.40	4.71
Subtraction	T1	780	11.99	4.50	789	16.36	5.23	769	19.20	5.12	793	22.12	5.09	837	25.34	5.15
	T2	733	13.64	4.85	769	17.77	5.34	823	20.68	5.09	824	23.67	5.34	848	26.20	5.21
	T3	703	14.82	5.34	686	18.51	5.44	718	21.35	5.21	709	24.12	5.17	764	26.24	5.41
Multiplication	T1	-	-	-	787	16.18	4.64	769	19.46	5.29	793	22.28	5.51	837	25.12	5.73
	T2	-	-	-	769	18.20	4.98	823	20.65	5.34	823	23.33	5.71	848	26.49	5.86
	T3	-	-	-	685	18.56	5.20	718	21.13	5.43	709	23.72	5.66	764	25.81	6.07
Division	T1	-	-	-	767	7.98	5.29	769	12.67	5.97	793	16.53	6.63	837	20.90	7.21
	T2	-	-	-	767	10.93	5.72	823	14.86	6.49	822	18.45	6.79	848	22.54	7.32
	T3	-	-	-	682	11.81	6.11	717	16.18	6.43	708	19.48	6.85	764	22.27	7.89
Visual-spatial WM		754	.56	.18	745	.65	.17	744	.71	.15	787	.74	.13	800	.77	.13
Verbal WM		727	.46	.15	641	.51	.14	725	.56	.12	698	.58	.13	708	.62	.13

T1 = beginning of school year, T2 = mid school year, T3 = end of school year, WM = working memory

### Multigroup latent growth curve modeling

#### Overall growth models of math fluency

In step 1, in all four overall univariate multilevel latent growth models (i.e., addition, subtraction, multiplication, and division), we found a non-significant covariance between intercept and slope. Removal of the non-significant covariances did not result in a significant decrement, Δχ^2^ = 1.68, 0.90, 3.49, 0.06, respectively, all df = 1, all *p* = n.s., and provided good to acceptable fit indices for the multilevel latent growth models for addition, χ^2^(2) = 8.89, *p* = .012, CFI = .999, TLI = .999, RMSEA = .03, subtraction, χ^2^(2) = 16.32, *p* = .000, CFI = .999, TLI = .998, RMSEA = .04, multiplication χ^2^(2) = 32.07, *p* = .000, CFI = .993, TLI = .989, RMSEA = .06, and division, χ^2^(2) = 39.71, *p* = .000, CFI = 1.000, TLI = .999 RMSEA = .07. Unstandardized estimates for intercepts and slopes within these models are presented in Table 3.

**Table 3:** Unstandardized estimates for Univariate Multilevel Latent Growth Models of math fluency for the total sample and for the grades separately

	Intercept		Slope	
Math domain	mean	variance	mean	variance
Addition				
Overall model	22.98***	37.11***	1.18***	1.02
Grade 2	16.15***	15.36***	1.21***	2.83***
Grade 3	19.89***	19.04***	1.23***	2.75***
Grade 4	23.32***	17.59***	1.43***	1.21*
Grade 5	26.26***	17.99***	1.13***	0
Grade 6	28.65***	16.33***	0.90***	0.70
Subtraction				
Overall model	20.24***	40.03***	1.09***	1.17*
Grade 2	13.46***	17.22***	1.50***	1.53***
Grade 3	17.64***	21.98***	1.21***	1.47***
Grade 4	20.45***	20.58***	1.10***	0.29
Grade 5	23.30***	21.13***	1.11***	0.34
Grade 6	25.90***	22.49***	0.56***	0
Multiplication				
Overall model	21.30***	37.82***	0.85***	1.43*
Grade 3	17.61***	18.38***	1.28***	1.63***
Grade 4	20.45***	22.87***	0.86***	1.20*
Grade 5	23.11***	26.25***	0.82***	0.92
Grade 6	25.74***	29.52***	0.47***	0.49
Division				
Overall model	15.91***	57.82***	1.51***	2.06*
Grade 3	10.22***	23.25***	2.08***	3.45***
Grade 4	14.60***	31.81***	1.68***	2.14**
Grade 5	18.11***	37.97***	1.56***	2.10**
Grade 6	21.86***	48.51***	0.86***	0.98

*** *p* < .001, ** *p* < .01, * *p* < .05

#### Multigroup growth models of math fluency

In step 2, multigroup analysis revealed a negative and non-significant slope variance for grade 5 in the domain of addition and for grade 6 in the domain of subtraction. Therefore, these slope variances were fixed to zero (Muthen & Asparouhov, 2011). For all other domains, no such problems arose. Multigroup estimates (see Table 3) show that the intercept means differ significantly from zero in each grade for addition, subtraction, multiplication, and division. Wald tests show that the mean intercept increases significantly (after Holm’s correction) between the lowest and highest grade, *Wald*(1) ranges from 275.39 to 1221.73, all *p*-values < .001, indicating that children in higher grades perform better in all four domains. In addition, the significant intercept variances indicate that within in each grade, there are significant individual differences between children in their level of performance on addition, subtraction, multiplication, and division problems. In general, individual variation in the level of performance seems to increase in higher grades in each domain. Wald tests show that this increase in variation is significant (after Holm’s correction) when comparing the lowest to the highest grade in subtraction, *Wald*(1) = 6.96, *p* < .01, multiplication, *Wald*(1) = 26.98, *p* < .001, and division, *Wald*(1) = 54.99, *p* < .001, but not in addition, *Wald*(1) = 0.26, *p* = .61.

Furthermore, we found the mean slope for all four math domains differed significantly from zero in every grade. So, on average, children in each grade show a significant mean increase in their ability to add, subtract, multiply, or divide throughout the school year. When we look at differences between grades in slope means (see Table 3), we can see that the mean rate of change during the school year decreases with age, and is lowest in grade 6 in all of the domains. This decrease from grade 2 or 3 to grade 6 is significant (after Holm’s correction) in the domains of subtraction, multiplication, and division, *Wald*(1) ranges from 20.47 to 28.08, all *p*-values < .001, but not in addition, *Wald*(1) = 2.38, *p* = .12. Slope variances are significant in the domains of addition and subtraction in children within lower grades (i.e., 2, 3, and 4), indicating that they show significant individual differences in their rate of change, but not for children in higher grades (i.e., 4, 5, and 6). In the domains of multiplication and division, however, children within each grade show significant individual differences in their rate of change, although the variation does seem to become smaller in higher grades. Overall, slope variance seems to decline in higher grades in all domains. Wald tests show that these differences are significant (after Holm’s correction) between the lowest and highest grades in addition, *Wald*(1) = 8.20, *p* < .01, subtraction, *Wald*(1) = 13.21, *p* < .001, and division, *Wald*(1) = 15.33, *p* < .001, but not in multiplication after Holm’s correction, *Wald*(1) = 6.49, *p* = .019 > Holm’s corrected *p*-value of .016.

#### Overall predictive value of visual-spatial and verbal working memory for math fluency

In step 3, adding visual-spatial and verbal working memory as predictors resulted in good fit indices for the models in the domains of addition, χ^2^(4) = 13.85, *p* = .008, CFI = 1.000, TLI = .999, RMSEA = .02, subtraction, χ^2^(4) = 24.43, *p* = .000, CFI = .998, TLI = .996, RMSEA = .03, multiplication, χ^2^(4) = 49.47, *p* = .000, CFI = .993, TLI = .985, RMSEA = .05, and division, χ^2^(4) = 58.40, *p* = .000, CFI = .996, TLI = .991, RMSEA = .05. Standardized estimates are presented in Table 4. From this table, we see that in the overall models, visual-spatial and verbal working memory explained a significant amount of variation in the intercept in each math domain. However, when variation in the slope was significant it could not be significantly explained by visual-spatial and verbal working memory.

**Table 4:** Standardized estimates and explained variance (*R* ^2^) of visual-spatial (VS) working memory and verbal (VE) working memory from multilevel regression model to predict intercept and slope in math fluency, and difference between standardized estimates (*Z*_*H*_)

	intercept				slope		
	VS	VE	*R* ^*2*^	*Z*_*H*_^a^	VS	VE	*R* ^*2*^
Addition							
Overall model	.33***	.31***	.31***	-	.02	-.00	.00
Grade 2	.14**	.14*	.05*	0.0	.14**	-.00	.02
Grade 3	.18***	.19***	.08***	-0.2	-.01	-.01	.00
Grade 4	.16**	.16**	.07*	0.0	.06	.04	.01
Grade 5	.10	.24***	.08**	-3.8***	-	-	-
Grade 6	-.01	.28***	.07**	-8.2***	-	-	-
Subtraction							
Overall model	.33***	.33***	.32***	-	-.14	-.00	.02
Grade 2	.14**	.20**	.07**	-1.4	.03	.16*	.03
Grade 3	.22***	.22***	.12***	0.0	-.02	.04	.00
Grade 4	.18**	.21***	.11**	-0.8	-	-	-
Grade 5	.08	.25***	.09***	-4.6***	-	-	-
Grade 6	.05	.29***	.10**	-6.8***	-	-	-
Multiplication							
Overall model	.24***	.27***	.19***	-	-.05	-.00	.00
Grade 3	.18***	.10*	.05**	1.9	.09	-.01	.01
Grade 4	.10	.13*	.04	-0.8	-.09	.18	.03
Grade 5	.05	.17***	.04*	-3.2**	.14	-.07	.02
Grade 6	-.02	.24***	.05**	-7.3***	.02	.19	.04
Division							
Overall model	.28***	.30***	.26***	-	-.08	.03	.00
Grade 3	.22***	.15**	.09**	1.7	.04	.02	.00
Grade 4	.17***	.17***	.08**	0.0	.03	.11	.01
Grade 5	.13*	.21***	.08**	-2.5*	.01	.05	.00
Grade 6	.06	.29***	.10***	-6.6***	-.02	.23	.05

Two-sided *p*-values: *** *p* < .001, ** *p* < .01, * *p* < .05

^a^Holm’s correction for multiple comparisons has been applied

#### Multigroup predictive value of visual-spatial and verbal working memory for math fluency

In step 4, we again performed multigroup analysis. Standardized estimates for each grade are presented in Table 4. For the domain of addition, visual-spatial working memory predicted level of performance in grades 2, 3, 4, and 5, but not grade 6. Verbal working memory on the other hand predicted level of addition performance in each grade (2-6). For subtraction, visual-spatial working memory predicted performance level in grades 2, 3, and 4, but not grades 5 and 6, while verbal working memory predicted performance level in each grade (2–6). With regard to multiplication, visual-spatial working memory predicted level of performance in grade 3, but not grades 4, 5, and 6. Verbal working memory predicted level of multiplication performance in each grade (3–6). With regard to division, visual-spatial working memory predicted performance level in grades 3, 4, and 5, but not grade 6. Verbal working memory predicted performance level in division in each grade (3–6). The amount of variation in level of performance explained by visual-spatial and verbal working memory combined is significant in each grade and each domain, except for multiplication in grade 4. The amount of variation in the rate of developmental change in math fluency explained by visual-spatial or verbal working memory was not significant in any of the domains or grades (see Table 4).

Inspection of Table 4 reveals a pattern with regard to differences in the predictive value of visual-spatial working memory and verbal working memory within grades. Within grades 2–4, visual-spatial and verbal working memory were equally predictive for performance in all four math domains (see Table 4 for *Z*_*H*_-scores for within grade comparisons). In grades 5 and 6, however, the predictive value of verbal working memory becomes significantly larger than the predictive value of visual-spatial working memory for level of performance in all four domains.

**Fig. 1:**
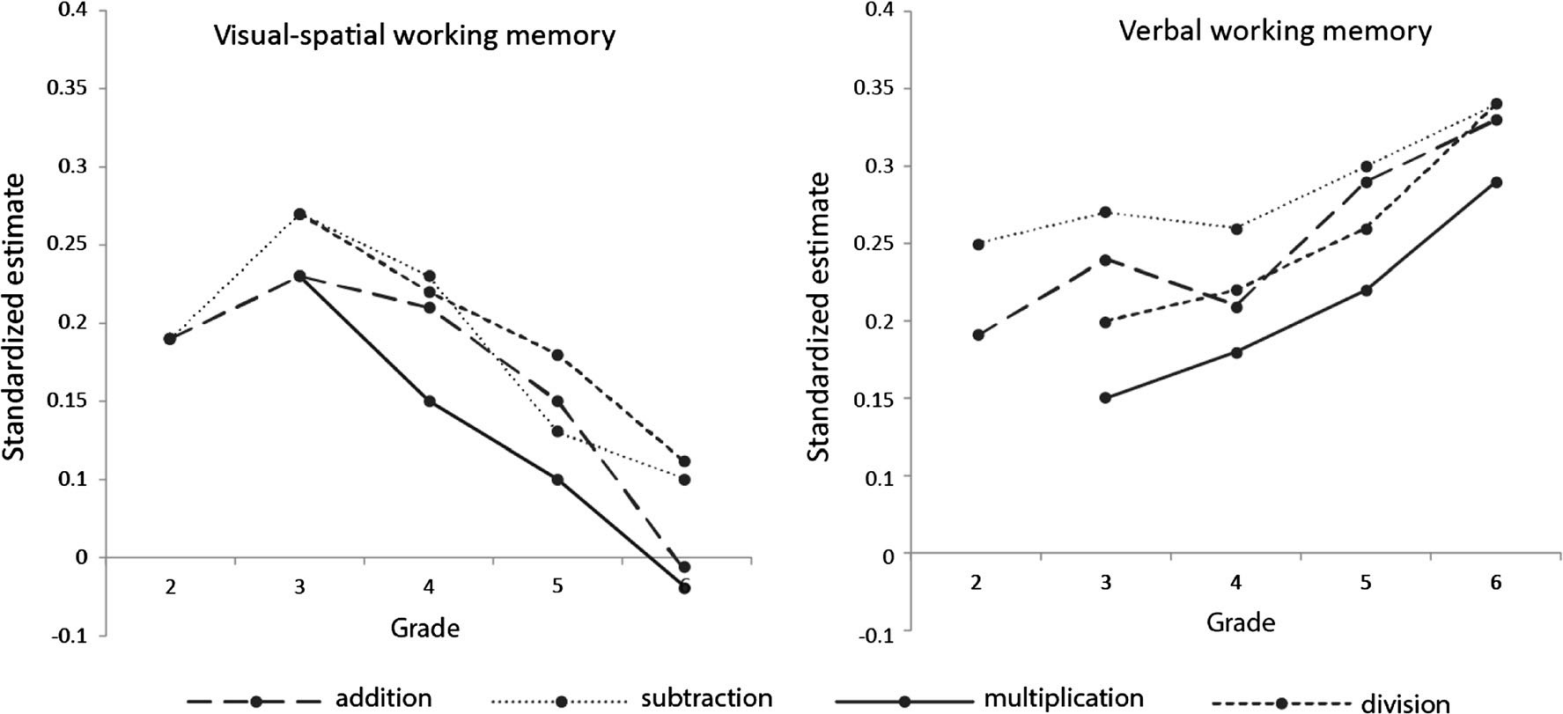
The predictive value of visual-spatial (left) and verbal (right) working memory for level of performance in the different grades per mathematical domain

Inspection of Table 4 also reveals several patterns of differences between grades. The predictive value of visual-spatial working memory for level of addition and subtraction performance increases somewhat from grade 2 to grade 3, but then declines from grade 3 onwards for all math domains, with standard estimates becoming smaller and significance levels becoming higher or becoming non-significant (see Fig. 1 for a visual presentation of these results). The decline between grade 2 and grade 6 in addition is significant, but in subtraction this is not significant (see Table 5 for *Z*-scores from between grade comparisons), but this is not surprising because of the initial increase from grade 2 to grade 3. Therefore, in the domains of addition and subtraction we also compared standardized estimates between grades 3 and 6. Indeed, from grade 3 to grade 6 the declines are significant in each domain (see Table 5). The predictive value of verbal working memory for level of performance shows an opposite pattern, with standard estimates becoming larger and significance levels becoming lower from grade 2 to grade 6 for addition and subtraction, or from grade 3 to grade 6 for multiplication and division. Although the increases from the lowest to the highest grade are significant initially, some of the results fail to be significant after Holm’s correction for multiple comparisons (see Table 5) is applied. Interestingly, the predictive value of verbal working memory shows a small dip in grade 4 for the domains of subtraction and addition (see Fig. 1).

**Table 5:** Z-scores for comparison between lowest and highest grades of standardized estimates indicating predictive value of working memory for mathematical fluency in four domains

	Grade 2 versus 6	Grade 3 versus 6
Visual-spatial WM		
Addition	3.2**	4.0***
Subtraction	1.9	3.6***
Multiplication	-	4.2***
Division	-	3.4***
Verbal WM		
Addition	-3.1**	-
Subtraction	-2.0^a^	-
Multiplication	-	-3.0**
Division	-	-3.1**

Two-sided *p*-values: *** *p* < .001, ** *p* < .01, ^a^ *p*-value of .045 > Holm’s corrected *p*-value .025

#### Growth rate in math fluency

Visual-spatial working memory significantly predicted children’s rate of change in performance of addition in grade 2, but not in any of the other grades or math domains. Verbal working memory was a significant predictor of grade 2 children’s rate of change in subtraction performance, but not in any of the other grades or domains. However, although standardized estimates of the relationships mentioned above were significant, the variance explained in the slope failed to reach significance.

#### Covariance between visual-spatial and verbal working memory

The covariance between visual-spatial and verbal working memory is significant in each grade (all *p*-values < .001), but the strength of the relationship seems to increase in higher grades, standard estimate = .22, .25, .41, .39, .41 in grades 2, 3, 4, 5, and 6 respectively. Fisher’s *r*-to-*z* transformation shows that this increase from grade 2 to 6 is significant, Z = -4.4, *p* < .001 (after Holm’s correction).

## Discussion

The aim of this article was to examine age-related changes in the predictive value of visual-spatial and verbal working memory for level and developmental change in mathematics performance on addition, subtraction, multiplication, and division problems. The results indicate that visual-spatial and verbal working memory predict individual differences in children’s level of performance in each math domain, but are not predictive of individual differences in growth rate over the school year.

Our results provide strong evidence for the developmental explanation. Overall, the predictive value of visual-spatial working memory for level of performance increases somewhat from grade 2 to grade 3 for addition and subtraction, but declines from grade 3 onwards in all math domains. Visual-spatial working memory is no longer a significant predictor of addition, subtraction, and multiplication in grade 5, nor of division in grade 6. The predictive value of verbal working memory, on the other hand, increases from grade 2 onwards in each math domain, except for a temporary drop in grade 4 for addition and subtraction. Until grade 4, visual-spatial and verbal working memory are equally strong predictors of performance, but in grades 5 and 6, verbal working memory takes over. These results are in line with previous studies indicating an age-related shift from visual-spatial representations and strategies to verbal representations and strategies (De Smedt et al., 2009; Geary et al., 2004). It is sometimes assumed that direct retrieval of math facts requires less working memory capacity (Ackerman, 1988; Ackerman & Gianciolo, 2000; Geary et al., 2004), and it has been shown that working memory is less involved in arithmetic as children grow older, presumably as a result of changes in strategy efficiency and selection (Imbo & Vandierendonck, 2007). However, in our study, we found no declining influence of working memory with age. In contrast, the influence of verbal working memory increased over age and the percentage of variance explained by working memory remained stable from grade 2 to grade 6. However, this could be a result of the speeded nature of the arithmetic test that was used. Although children all solved the same math problems, children who solve math problems more efficiently (e.g., by direct retrieval) will progress further through the test, encountering increasingly difficult problems, which probably elicit more procedural strategies.

Our results do not provide evidence for differential contributions from visual-spatial and verbal working memory to the different math domains. The changes in the relationships across age between the two measures of working memory and math performance are similar in all four math domains. These results are in contrast with the findings of Van der Ven et al. (2013), who did not find a decreasing age-trend for the predictive value of visual-spatial working memory for multiplication and division. Based on our results, we assume that the procedural strategies that are used in all domains impose a load on visual-spatial working memory initially, but are replaced by verbal strategies and retrieval. Furthermore, there are no clear indications that the timing of such replacement differs between domains. An exploratory factor analysis revealed that all four domains loaded onto one factor. Although this may indicate that all four domains reflect math fluency, children will not have memorized all the problems they solved, and the different types of calculations may require different abilities.

We found some evidence for the novelty explanation. There was an initial increase from grade 2 to grade 3 in the predictive value of visual-spatial working memory for performance in addition and subtraction. However, we found no peaks in the relationship between visual-spatial working memory and performance in the grades in which that domain was introduced. Although the grade 4 ‘dip’ in the predictive value of verbal working memory for performance in addition and subtraction was non-significant, it is clear that the predictive value was stable between grades 3 and 5. However, apparently in grade 4, addition and subtraction facts are automatized to a certain extent, where individual differences in working memory play a smaller role. Increased attention for rote learning may temporarily reduce the influence of individual differences in verbal working memory (Geary et al., 2004; Imbo & Vandierendonck, 2007; Tronsky, 2005). In fact, the predictive value of both visual-spatial and verbal working memory was lowest in multiplication, which could reflect the fact that verbal rote memorization is applied more often for multiplication than for any of the other domains in the school curriculum. Indeed, the Dutch government has set attainment targets for primary mathematics education, which have been formulated into indications for (the timing of) specific content and activities by the Netherlands Institute for Curriculum Development (Ministerie van Onderwijs, Cultuur en Wetenschap, 2006). In grades 3 and 4, increased attention for learning math facts is advised. However, the fact that this ‘dip’ was not found in multiplication and division may indicate that these domains are less automatized in grade 4, perhaps because they are introduced later. The data also suggest that automatization for addition and subtraction waned in the higher grades, since the predictive value of verbal working memory increases again in grades 5 and 6.

Both visual-spatial and verbal working memory did not predict individual differences in the rate of developmental change in math performance. This is in contrast with previous studies, in which working memory was found to be predictive of mathematics achievement growth during primary school (Hecht, Torgesen, Wagner, & Rashotte, 2001; Swanson, Jerman, & Zheng, 2008). However, these studies modeled growth over longer periods of time (e.g., first to fifth grade), while in our study growth within one school year was modeled. In some domains, such as addition and subtraction, older children did not differ in their rate of change. However, in other domains, children did differ significantly in their rate of change, but perhaps that individual variation in the rate of change was too small over such a short period of time to find a relationship with working memory. It might also be possible that while individual variation in the level of mathematics performance is influenced largely by individual differences in cognitive processes, developmental change during the school year(s) is influenced mainly by classroom factors, such as the quality of instruction given by the teacher or the time spent on rote learning in a classroom (Crosnoe et al., 2010). In line with this, a study by Geary et al., (1996) showed that Chinese students showed more improvement on direct retrieval strategies, which in large part could be explained by a larger quantity of instruction and exposure to math in Chinese schools.

Another interesting finding is that the strength of the relationship between visual-spatial and verbal working memory increased with age. Previous studies found mixed results with regard to this issue (Alloway, Gathercole, & Pickering, 2006; Gathercole, Pickering, Ambridge, & Wearing, 2004). Whereas Alloway and colleagues (2006) did find the strength of the association between verbal and visual-spatial short term memory to increase with age, Gathercole et al. (2004) did not. Alloway et al. (2006) believe their findings indicate that older children increasingly use verbal labels to recode even visual information, although the other way around could also be argued – that children visualize verbal information during working memory tasks.

Interestingly, while individual differences in level of performance increased over grades in all domains, except addition, individual differences in rate of growth decreased over grades in all four domains. So our data indicates that students’ arithmetical problem-solving is more similar to one another at the beginning of primary school, and that individual differences become larger over the school years with some students showing faster rate of growth than others. Despite such individual differences, however, rate of growth becomes more comparable between children over the school years, indicating that individual differences at the end of primary school become more stable. These findings may reflect developmental processes, but also show how changes in math education are deployed throughout primary school grades. It is known that early numeracy, during the first primary school years, provides an important base for later mathematical learning and thinking (Siegler, 2009; Toll & Van Luit, 2014). Perhaps more attention is paid to individual differences and remediation at the beginning of primary school, while in higher grades individual differences may be considered more crystallized and taken as a given. However, since these comparisons are partially cross-sectional, it may well reflect increased awareness for tailoring education to individual differences and needs. Perhaps children in grade 6 started primary school at a time when it was still more standardized practice to bring all students to the same level of performance.

This study has several strengths and weaknesses that are worth mentioning. The strengths are the large sample size and inclusion of children from a wide range of grades, making it possible to systematically examine the changes in predictive value of working memory for mathematics throughout primary school. A limitation of this study is that visual-spatial and verbal working memory were each assessed with only one test. Also, although we tried to minimize the influence of reading ability, it is possible that individual differences in reading ability influenced performance on the Monkey game. Future studies may use several tests for each component, and use a latent variable approach for more reliable measurement. Another limitation is that only a speeded arithmetic test was used, which is a limited measure of mathematics performance. This timed element of the test may have increased the influence of processing speed, which also plays a role in performance on working memory tests (Conway, Cowan, Bunting, Therriault, & Minkoff, 2002), and may therefore have confounded associations between the two. On the other hand, our results are similar to findings from other studies in which no timed math tests were used (De Smedt et al., 2009; Geary et al., 2004), and we would expect processing speed to influence both visual-spatial and verbal working memory tasks. Therefore, processing speed as a possible confounding factor cannot account for the age-related shift that was found. Future studies should also include other types of math tests, in which a broader range of mathematics abilities are assessed. Finally, although we used a longitudinal design, the period of the study was too short to find a large enough variation in the rate of growth over the school year. In future research, longitudinal measures over longer periods of time (e.g., several school years) are recommended.
